# Trade in Zambian Edible Orchids—DNA Barcoding Reveals the Use of Unexpected Orchid Taxa for *Chikanda*

**DOI:** 10.3390/genes9120595

**Published:** 2018-11-30

**Authors:** Sarina Veldman, Seol-Jong Kim, Tinde R. van Andel, Maria Bello Font, Ruth E. Bone, Benny Bytebier, David Chuba, Barbara Gravendeel, Florent Martos, Geophat Mpatwa, Grace Ngugi, Royd Vinya, Nicholas Wightman, Kazutoma Yokoya, Hugo J. de Boer

**Affiliations:** 1 Department of Organismal Biology, Systematic Biology, Uppsala University, Norbyvägen 18D, 75236 Uppsala, Sweden; seoljong.kim@gmail.com (S.-J.K.); h.d.boer@nhm.uio.no (H.J.d.B.); 2 Naturalis Biodiversity Center, P.O. Box 9517, 2300 RA Leiden, The Netherlands; tinde.vanAndel@naturalis.nl (T.R.v.A.); barbara.gravendeel@naturalis.nl (B.G.); 3 Natural History Museum, University of Oslo, Postboks 1172, Blindern, 0318 Oslo, Norway; mariabellofont@hotmail.com; 4 Royal Botanic Gardens, Kew, Richmond, Surrey TW9 3AB, UK; R.Bone@kew.org (R.E.B.); K.Yokoya@kew.org (K.Y.); 5 Bews Herbarium, School of Life Sciences, University of KwaZulu-Natal, Pr. Bag X01, Scottsville 3209, South Africa; Bytebier@ukzn.ac.za (B.B.); florentmartos@gmail.com (F.M.); grace.ngugi@yahoo.com (G.N.); 6 Department of Biological Sciences, University of Zambia, Box 32379 Lusaka, Zambia; david.chuba@unza.zm; 7 Institute of Biology Leiden, Leiden University, P.O. Box 9505, 2300 RA Leiden, The Netherlands; 8 University of Applied Sciences Leiden, Zernikedreef 11, 2333 CK Leiden, The Netherlands; 9 Institut de Systématique, Evolution, Biodiversité (ISYEB), Muséum national d’histoire naturelle, CNRS, Sorbonne Université, EPHE, CP50, 45 rue Buffon 75005 Paris, France; 10 School of Natural Resources, The Copperbelt University, PO Box 21692 Kitwe, Zambia; gmpatwa@gmail.com (G.M.); royd.vinya@gmail.com (R.V.); 11 East African Herbarium, National Museums of Kenya, P.O. Box 40658-00100 Nairobi, Kenya; 12 Homegarden Landscape Consultants Ltd., P/Bag 30C, Chilanga, Lusaka, Zambia; homegarden.nicholas@gmail.com

**Keywords:** CITES, chikanda, conservation, DNA barcoding, orchids, species identification

## Abstract

In Zambia, wild edible terrestrial orchids are used to produce a local delicacy called *chikanda*, which has become increasingly popular throughout the country. Commercialization puts orchid populations in Zambia and neighbouring countries at risk of overharvesting. Hitherto, no study has documented which orchid species are traded on local markets, as orchid tubers are difficult to identify morphologically. In this study, the core land-plant DNA barcoding markers *rbcL* and *matK* were used in combination with nrITS to determine which species were sold in Zambian markets. Eighty-two interviews were conducted to determine harvesting areas, as well as possible sustainability concerns. By using nrITS DNA barcoding, a total of 16 orchid species in six different genera could be identified. Both *rbcL* and *matK* proved suitable to identify the tubers up to the genus or family level. *Disa robusta*, *Platycoryne crocea* and *Satyrium buchananii* were identified most frequently and three previously undocumented species were encountered on the market. Few orchid species are currently listed on the global International Union for the Conservation of Nature (IUCN) Red List. Local orchid populations and endemic species could be at risk of overharvesting due to the intensive and indiscriminate harvesting of *chikanda* orchids, and we therefore encourage increased conservation assessment of terrestrial African orchids.

## 1. Introduction

Terrestrial orchids have been used for medicinal and culinary purposes for centuries [1], with the most notable example being the use of orchid tubers to make *salep*, a traditional Turkish creamy drink or ice cream, consumed in Asia Minor and several countries on the Balkan peninsula [1,2,3,4]. In south-eastern Africa, terrestrial orchid tubers are mixed with peanut flour, salt, baking soda and chili powder to make a traditional Zambian meat-like cake known as *chikanda* or African polony [5,6,7]. Although initially not highly regarded [8], chikanda has more recently become popular throughout the country. It is sold as a snack along the streets, on markets, in supermarkets and on the menu of high-end restaurants [9] and recipes; in addition, cooking tutorial videos can be found online [10]. Orchids used for chikanda are harvested exclusively from the wild, and although it is unlikely that traditional village consumption poses a serious threat to orchid populations, the increased popularity and subsequent commercialization of chikanda has led to the exhaustion of Zambian orchid resources [7]. Collecting tubers means the end of a perennial and generally long-lived orchid, since the entire plant is removed in the harvesting process.

Soweto market in the Zambian capital Lusaka is the hub of the chikanda trade. Surveys performed on this market have shown that a large part of the chikanda tubers sold are sourced from Tanzania and that Zambian chikanda orchids are collected from the Luwingu, Mporokoso and Kasama districts in the Northern Province and Serenje in the Central province [6,7]. According to local chikanda vendors, another region with a flourishing chikanda trade is the Kitwe region in the Copperbelt Province, but so far, no surveys have been performed there. Despite international legislation initiated by the Convention on International Trade in Endangered Species of Wild Fauna and Flora (CITES) banning cross-border trade, an estimated 2–4 million orchid tubers are transported annually from Tanzania to Zambia [7,11]. Import from the surrounding countries of Angola, Democratic Republic of the Congo (DRC), Malawi and Mozambique is also documented [7,9]. Orchid species originally reported as ingredients for chikanda are *Disa robusta* N.E.Br. and *Satyrium buchananii* Schltr. [11,12], whereas at least 32 species belonging to the genera *Brachycorythis*, *Disa*, *Eulophia*, *Habenaria, Roeperocharis* and *Satyrium* were recently suggested to be used for chikanda production based on collections in the field [11,13,14,15,16,17,18] and one metabarcoding study of ready-made chikanda cakes [18]. To date, however, no study has identified the orchids traded at the local markets, since the tubers lack sufficient morphological characters for taxonomic identification to species level [7,18]. Local classification systems categorize the tubers based on texture, harvesting locality, soil color and phenology, but these are not likely to be congruent with scientific classifications [5,14].

Knowing which orchid species are currently being collected for the expanding chikanda trade enables the identification of species susceptible to overharvesting and can inform conservation planning. Molecular methods such as DNA barcoding can be applied to identify samples when morphological diagnostic characters for identification are lacking [19]. DNA barcoding and metabarcoding has proven to be effective in the authentication of commercial wood species (Jiao, 2018), medicinal plants [20] and salep-producing orchids on Iranian markets [4]. The analysis of ingredients in Tanzanian chikanda cake with DNA metabarcoding revealed the presence of 21 different orchid species [18], but a DNA barcoding approach has not yet been applied to individual orchid tubers used to make this product. The aim of this study was to test to what extent the use of standard molecular markers yields robust identification of chikanda orchid tubers traded on Zambian markets. Molecular identification can enable the mapping of the harvesting and trade of specific Zambian orchid species and facilitate the identification and implementation of targeted conservation strategies. Within that framework, this study also aimed to identify conservation issues associated with the chikanda trade, and addresses the following questions: (1) Which species are used for chikanda production in the Lusaka and Kitwe districts of Zambia, and what is their geographic origin? (2) Can chikanda tubers be identified up to species level using DNA barcoding? (3) How do local classification systems relate to scientific species concepts? (4) What are the main conservation issues associated with chikanda trade in the Lusaka and Kitwe districts?

## 2. Materials and Methods

### 2.1. Interviews and Sample Collection

**Figure 1:**
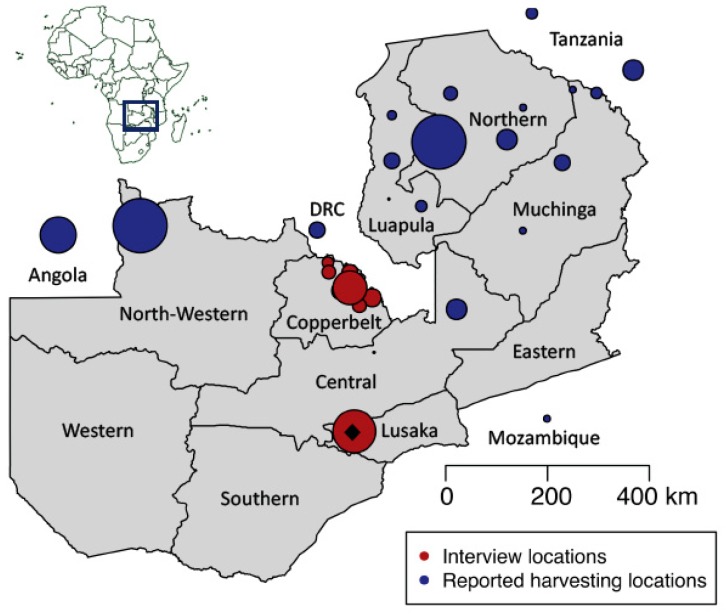
Map of Zambia with an overview of interview localities and reported provenance of the chikanda tubers. Dot size corresponds to the number of informants.

Fieldwork in Zambia was conducted in 2016 in the Kitwe, Kalulushi, Luanshya, Ndola, Mufulira, Chingola and Chililabomwe districts of the Copperbelt Province, the Kapiri Mposhi and Serenje districts in the Central Province, and in the capital Lusaka (Figure 1). Semi-structured interviews were conducted with harvesters, middlemen and vendors to obtain insight into chikanda commercialization, harvesting times, preferences and availability. The questionnaires consisted of three sections, one on informant and interview characteristics, one with general questions about chikanda posed to all informants, and a third section with questions more specifically designed for each interviewee category: harvester, middleman and vendor. All research was conducted in accordance with the International Society of Ethnobiology Code of Ethics [21]. Ethical clearance was obtained from the Humanities and Social Science Research Ethics Committee of the University of Zambia. The interviews were performed in English or Bemba, with a translator affiliated with the University of Zambia and the Copperbelt University. Informants were selected using the snowball technique [22] by asking people whether they could direct us to people harvesting or selling chikanda. All informants were provided with information about the study and signed a prior informed consent sheet. Fieldwork took place during June and July, the peak season for chikanda [7], to ensure the collection of both fresh and dried chikanda tubers on the market and in the field. A collection was made each time a specific vernacular chikanda type was bought from a specific vendor, and assigned a collection number (SJK1, SJK2, etc.). Each individual tuber within the collection received a subsample number within that collection (SJK1.1, SJK1.2, SJK 1.16, etc.). The fresh tubers were sliced and stored with silica gel in plastic bags. All chikanda samples were brought to Sweden under a CITES inter-institutional exchange agreement between the University of Zambia (ZM001) and the Botany Section of the Evolutionary Museum in the Evolutionary Biology Center in Uppsala (SE009). Export permission was obtained from the Zambian CBD and Nagoya Protocol focal point at the Ministry of Natural Resources and Environmental Protection. Upon arrival in Sweden, some of the chikanda tubers had sprouted. Those were transferred to the Uppsala Botanical Garden for cultivation and subsequent sampling of fresh leaf tissue for DNA barcoding as well as morphological identification.

### 2.2. Reference Taxon Sampling

Herbarium specimens were collected with associated silica-dried material for DNA extraction and spirit collections during fieldwork in Zambia in January and February 2017. All material was collected and exchanged in accordance with national and international legislation. The collections were deposited at the Division of Forest Research (Kitwe, Zambia) and RBG Kew (UK) and field identifications verified at the Bews Herbarium (South Africa). A total of 94 novel Orchidaceae reference vouchers were collected for this study, representing 4 *Brachycorythis*, 9 *Disa*, 16 *Habenaria*, 6 *Satyrium* species and 26 species of other orchid genera. Voucher specimens of all taxa sampled are listed in Table S1 (Supplementary Materials). In addition, 88 nrITS, 71 *matK* and 45 *rbcL Habenaria* sequences generated for a forthcoming phylogenetic study [21], and 510 nrITS, 522 *matK* and 213 *rbcL* sequences corresponding to 311, 325 and 100 taxa in the previously mentioned orchid genera downloaded from GenBank were included in the reference database.

### 2.3. From Sample to Sequence

Out of the 1284 individual tubers in 48 different sample collections, 304 samples were selected for DNA extraction. To assess the orchid species present in each collection, a few tubers per sample (2–8) were extracted if the sample was morphologically homogenous, whereas more (6–33) were selected if the sample seemed diverse. In the selection process, we aimed to select tubers of different shapes and sizes to cover the potential species diversity in each collection. Since the selection appeared to be representative for each collection, it was not deemed necessary to extract DNA from each individual tuber in all market collections. DNA was extracted using a CTAB protocol [23] modified with 3 to 5 extra washing steps with STE buffer (0.25 M sucrose, 0.03 M Tris, 0.05 M EDTA) [4,24] to reduce the gelatinization effect of the large amount of polysaccharides in the starch-rich orchid tubers. Total DNA was stored in 70–100 μL 10 mM Tris-HCl buffer, pH 8.0. DNA concentration was measured with a Qubit 3.0 fluorometer (Thermo Fisher Scientific, Oakwood, GA, USA). The core land plant barcoding markers *rbcL* and *matK* were amplified using the primers and protocols described in Kress et al. [25] and Dunning and Savolainen [23] respectively. The reactions were performed in a total reaction volume of 25 μL with 14.725 μL ddH_2_O, 2.5 μL DreamTaq Buffer (Thermo Fisher Scientific, Oakwood, GA, USA), 0.5 μL 25 mM dNTP, 0.65 μL 2% bovine serum album (BSA), 0.125 μL DreamTaq Polymerase, 2.5 μL 5 pmol forward and reverse primer and 1.5 μL template DNA. Nuclear ribosomal ITS was amplified using the Sun et al. [26] primers and protocol in a total reaction volume of 25 μL containing 15.25 μL ddH_2_O, 2.5 μL DreamTaq Buffer (Thermo Fisher Scientific), 0.5 μL 25 mM dNTP, 0.125 μL 2% BSA, 0.125 μL DreamTaq Polymerase (Thermo Fisher Scientific), 2.5 μL 5 pmol forward and reverse primer and 1.5 μL template DNA. For ITS, an additional protocol was used with Q5 high-fidelity polymerase; reactions were performed in a total reaction volume of 23.5 μL including 10.875 μL ddH_2_O, 5 μL Q5 reaction buffer, 0.5 μL 25 mM dNTP, 5 μL Q5 GC enhancer, 0.125 μL Q5 high-fidelity polymerase, 1.5 μL 5 pmol forward and reverse primer and 0.5 μL template DNA. The PCR program for the ITS primers in combination with the Q5 polymerase was an initial heating step of 30s at 98 °C, 35 cycles of 10s 98 °C, 30s 56 °C, 30s 72 °C, and a final elongation of 2min at 72 °C. PCR products were cleaned using eight-times diluted ExoSAP (Thermo Fisher Scientific) and analysed on an ABI3730XL Sanger sequencer by Macrogen Europe (Amsterdam, The Netherlands). The obtained trace files were assembled using Pregap4 and Gap4 [27] as implemented in the Staden package [28]. Sequences shorter than 200 bp were discarded from the analysis and all sequences were deposited in NCBI GenBank (Table S2, Supplementary Materials). The NCBI BLAST algorithm was used to assess the identification of all the obtained query sequences using the Python BLAST tabular parser script (https://github.com/SLAment/Genomics/blob/master/BLAST/BLAST_tabularparser.py). Similarity scores, query coverage, expect value (E-value), and maximum identity percentage were calculated, and all the information of the top 5 hits were automatically mined and tabulated per marker (Tables S3–S5, Supplementary Materials) and summarized per individual tuber sample (Table S6, Supplementary Materials).

### 2.4. Phylogenetic Analysis and Species Identification

Alignments for nrITS, *matK* and *rbcL* were made using AliView [29], combing the query sequences and local reference databases consisting of sequences from NCBI GenBank and reference collections from fieldwork and unpublished data from collaborators [21]. Species identification was performed using the Bayesian implementation of the Poisson tree processes model (bPTP), as it has been shown to outperform the generalized mixed Yule coalescent (GMYC) approach as well as Operational Taxonomic Unit (OTU)-picking methods when evolutionary distances between species are short enough [30]. For all alignments, a maximum likelihood (ML) search for the best-scoring tree was performed using the RAxML web server [31] to generate input trees for species identification analysis using bPTP [30]. The default setting, GTRCATI, was used to implement the CAT approximation, and the final tree was evaluated using the traditional GTR model. The bPTP.py script setting was 100,000 Markov chain Monte Carlo (MCMC) iterations for all trees, a sampling interval thinning value of 100, a burn-in of 25%, and a random seed of 1234. No outgroup was defined. The generated convergence curve was visually confirmed. The description of each voucher is built up as follows: ‘sample #_BLAST search result_identification %_reported city/region of the origin_the country,’; for example: ‘SJK04.09_S.buchananii_97.893_Mwinilunga_Zambia’ (Figures S1–S3, Supplementary Materials). For the reference sequences, the accession number and species names are described for *Disa* and *Satyrium.* The reference *Habenaria* species and others are described as their species name and voucher (sample) number.

## 3. Results

### 3.1. Market Surveys and Interviews

We visited 25 markets in ten Zambian cities: seven cities in the Copperbelt Province, two in the Central Province, and one in Lusaka (Figure 1). Eighty-two persons involved in chikanda trade were interviewed, of which 8 were harvesters, 44 middlemen, 29 vendors and one informant was both harvester and middleman. The term ‘harvesters’ refers to people collecting chikanda in the field, ‘middlemen’ to people selling either dried or fresh chikanda orchid tubers on the market, and ‘vendors’ to informal street vendors selling ready-made chikanda cakes contained in a basket carried on the head. Since one of the harvesters also acted as middleman, in total, 83 individual interviews were conducted: 72 participants were female and 10 were male. The ages of the respondents varied between 18 and 78, with an average of 41 years. The majority of the respondents belonged to the Bemba tribe (62%), while the other respondents (38%) belonged to smaller ethnic groups. Most interviews were conducted in the Copperbelt Province (55), eight in the Central Province and 19 in Lusaka.

### 3.2. Local Classification System

**Figure 2:**
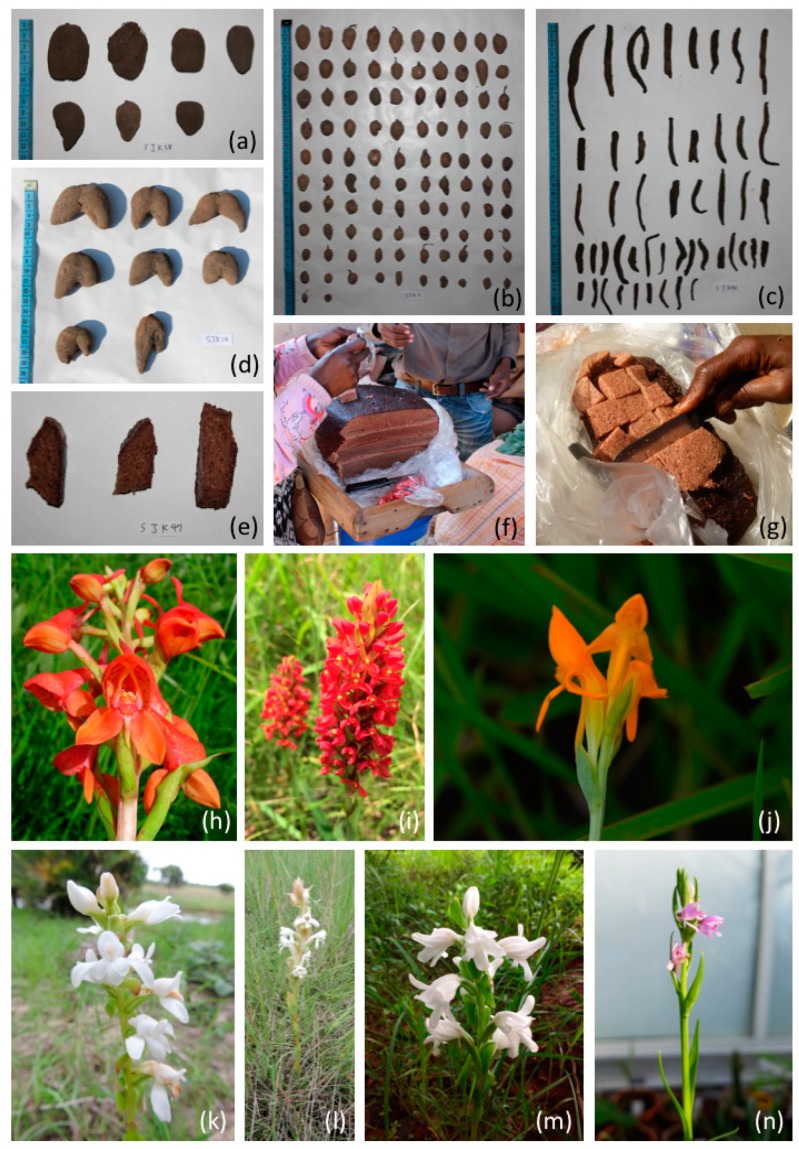
Chikanda tubers, cake and orchids. (**a**) Myala—real chikanda; (**b**) Mbwelenge—fake chikanda; (**c**) Mshilamshila—supposedly *Brachycorythis* sp.; (**d**) Mampanda. (**e**–**g**); Chikanda cake; (**h**) *Disa robusta*; (**i**) *Disa welwitschii*; (**j**) *Platycoryne crocea*; (**k**) *Satyrium carsonii*; (**l**) *Satyrium buchananii*; (**m**) *Satyrium kitimboense*; (**n**) *Brachycorythis* cf*. friesii*; Photographs (**a**–**g**) by Seol-Jong Kim, (**h**) by Robert v. Blittersdorff, (**i**,**k**,**l**) by Nicholas Wightman, (**j**) by Warren McCleland, (**m**) by Ruth E. Bone and (**n**) by Sarina Veldman.

Fifty-three different vernacular names for the various chikanda tuber types were recorded during the interviews. The most common way to distinguish between chikanda tubers was by using the terms original (*myala*) and fake (*mbwelenge* or *msekelele*). In some cases, it was the shape of the tuber that was used to differentiate between the different tubers: *mshilamshila* means root-like in Bemba and referred to the elongated, root-shaped tubers, whereas *mampanda* referred to the heart-shaped tubers. It also appeared common to use the origin of the tuber as a trade name: *mwinilunga*, *chozi*, *luwingu* and *kasama* are, for example, all Zambian city names, *sumbawanga* and *iringe* refer to Tanzanian cities (Sumbawanga and Iringa) and *angola* refers to one of the countries bordering Zambia. In some cases, the chikanda tubers were sold pre-mixed, whereas other vendors marketed the different types of tubers separately. The morphology and size of the tubers varied both within and between collections of a certain chikanda type. Tubers could be heart-shaped, rounded, egg-shaped to elongated and almost root-like. The largest tubers were the heart-shaped ones, which could be up to 5 cm long and 6 cm wide. The elongated tubers were up to 9 cm long with a maximum width of 2 cm. Harvesters, middlemen and vendors themselves indicated that they distinguish the tubers based on the size of the granules inside the tubers, which can be large or small, and in some tubers, a concentric ring was said to be present. Figure 2 illustrates the different tuber types and ready-made chikanda that were encountered on the local markets, as well as some of the orchid species producing these tubers.

### 3.3. Chikanda Trade and Availability

Many participants had a relatively long experience (an average of 13.5 years) in the chikanda trade. They indicated that they were asked by family, friends or neighbors to get involved in the chikanda business, or simply because it seemed to be a profitable industry. Some of the participants, especially harvesters, stated that they started collecting chikanda because the plants were easily accessible and were found growing close to their areas of residence. In general, people involved in the chikanda trade indicated that this work alone did not suffice to fully support themselves and their families, and these traders therefore supplemented their income by trading additional natural products such as fruits, maize, groundnuts, beans, mushrooms, snacks, herbs, and *kapenta* (a dried fish from Lake Tanganyika). In Lusaka, however, income generated with chikanda trade seemed to be sufficient for subsistence, and this suggests that there are significant local differences in profits generated in the chikanda business.

The chikanda trade was structured in several different ways. Some of the middlemen loaded the orchid tubers in trucks directly from the harvesting areas and brought them to larger cities such as Lusaka and Kitwe, whereas others relied on agents to gather a specified amount of chikanda tubers, which they paid for through the East-African mobile payment system M-Pesa and received as cargo from one of the local buses. In addition to the Tunduma and Nakonde markets that are the trade hubs between Tanzania and Zambia, other centers of trade were identified on the border with the DRC (Chililabombwe and Kasumbalesa) and Angola (Mwinilunga), where both chikanda tubers and ready-made chikanda cake were sold. An overview of all interview localities and the reported provenance of the chikanda tubers is given in Figure 1. Most of the participants indicated that chikanda plants are becoming locally rare. Middlemen emphasized the decrease of quantity, whereas harvesters were concerned about the decline in both quantity and quality (size and preferred chikanda type), which may also depend on the species. The participants from urban areas stated that access to chikanda tubers was managed by the chief of each tribe, who seasonally designated the harvestable *dambo* (wet meadow) within the chiefdom so that the collectors could maintain the quality of the harvests. Nevertheless, interviewed harvesters found in the dambo areas of Serenje (Central Province) claimed to be free to harvest tubers whenever available.

### 3.4. Molecular Identification of Traded Orchids

During the DNA extraction, many samples formed a thick jelly-like layer in the extraction tubes, despite repeated washing steps with STE buffer. This resulted in a very small water phase and is likely to have negatively influenced the downstream steps of the extraction process. The average DNA concentration of the chikanda tuber samples was 4.96 ng/μL, while the average DNA concentration from leaf samples from chikanda orchids was 28.5 ng/μL. Out of the 304 samples selected for DNA extraction, 232 samples produced detectable DNA. Amplification was attempted for each barcoding marker for all of the samples. A nrITS sequence was obtained for 159 samples, *rbcL* for 117 samples and *matK* only for 45 samples. Sequences from all three markers were obtained for 40 samples, sequences from two markers for 58 samples, and 55 samples only yielded sequences for a single marker. Analysis of the inter- and intraspecific variation of nrITS was performed for 141 sequences representing 124 *Disa* species, 73 sequences representing 59 *Habenaria* species and 110 sequences representing 67 *Satyrium* species. In the case of *matK* 135 sequences belonging to 122 *Disa* species were included, 507 *Habenaria* sequences belonging to 239 species and 116 sequences belonging to 60 *Satyrium* species. For *rbcL*, the available reference material was quite limited: 45 sequences for 37 *Habenaria* species, eight *Satyrium* sequences for two species, and four *Disa* sequences for four species.

**Figure 3:**
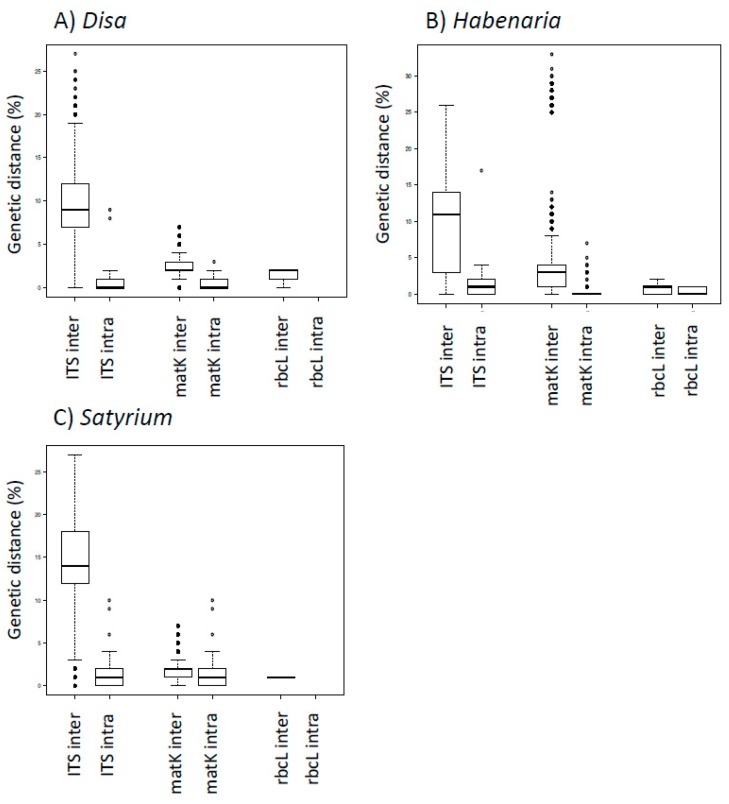
Boxplots showing the inter- and intraspecific variation for *Disa* (**a**)*, Habenaria* (**b**) and *Satyrium* (**c**) based on genetic diversity.

A graphical overview of the inter- and intraspecific variation per genus and per marker can be found in Figure 3. The interspecific variation for nrITS was significantly higher as compared with *matK* and *rbcL* in all three genera: on average, 10.2% in *Disa*, 9.51% in *Satyrium* and 8.75% in *Habenaria*. For *matK*, the interspecific variation was 2.39% for *Disa*, 2.79% for *Habenaria* and 1.39% for *Satyrium*. The intraspecific distances for nrITS and *matK* were, respectively, 1.37% and 0.61% for *Disa*, 1.98% and 0.36% for *Habenaria* and 1.45% and 0.48% for *Satyrium*, The limited reference sequences that were available for *rbcL* showed little pairwise interspecific distances (1.44% for *Disa*, 0.82% for *Habenaria* and 1.13% for *Satyrium*) and only allowed for the intraspecifc distance calculation of *Habenaria* (0.38%), indicating that *rbcL* is not suitable as a barcode for the species-level identification of chikanda orchids. The calculated thresholds were subsequently used to evaluate the identifications made with the online and standalone Basic Local Alignment Search Tool (BLAST).

In total, 15 orchid species were identified using DNA barcoding: *Brachycorythis* sp. SJK7, *Disa caffra* Bolus, *D. celata* Summerh., *D. robusta* N.E.Br., *D. satyriopsis* Kraenzl., *D. welwitschii* Rchb. f., *Disa* sp. SJK4.1., *Habenaria* sp. SJK31.15, *H.* aff. *helicoplectrum* Summerh., *Habenaria* cf. sp. DO112, *Platycoryne crocea* Rolfe, *Satyrium buchananii* Schltr., *S. carsonii* Rolfe, and *S. kitimboense* Kraenzl., as well as one species in an unidentified genus, which seems to be closely related to *Habenaria* based on the similarity-based BLAST identification. Additionally, one orchid that flowered from a sprouting tuber was identified based on morphology as *Brachycorythis* cf. *friesii* (Schltr.) Summerh.

bPTP analysis for *matK* and *rbcL* showed a lumping of several supposedly different species within one clade on several occasions (Figures S1 and S2, Supplementary Materials). In the case of nrITS, the bPTP outcome tree often reflected the expected species boundaries, although the posterior probabilities on the nodes were often too low to determine with confidence if species identification had been performed correctly (Figure S3, Supplementary Materials). Something that could be observed from both the nrITS BLAST identification, as well as the bPTP analysis, was that some of the identifications showed ambiguity: in collection SJK41, SJK44 and SJK46, some samples were identified unambiguously as *Platycoryne crocea*, whereas others showed a mix of *Habenaria* and *Platycoryne* top hits. The *matK* BLAST identification showed an unambiguous *P. crocea* identification for these samples, despite the presence of several *Habenaria* species in the reference database and despite the fact that *matK* shows a lower level of interspecific variation. A similar observation could be made for several *Disa* samples, but here a geographic pattern could be observed. The samples that could unambiguously be identified as *D. robusta* were all collected from Tanzania, the original source of the reference sequence. Some of the Zambian samples also showed *D. robusta* as the closest relative, but not with a high enough percentage identity match to confirm this identification. The bPTP result in this case shows a lumped and mixed clade with several *Disa* species and several other samples included, which confirms that sequence divergence is too limited to resolve the relationship. These samples were identified as *Disa* sp. 1, to reflect the fact that they all grouped together in the same clade. In other cases, such as for certain *Habenaria* species, we named the closely related sequences in the identification, whereas in this case the clade was too large to allow for this, since it contained the following sequences: *Disa engleriana* Kraenzl., *D. erubescens* Rendle., *D. miniata* Summerh., *D. ochrostachya* Rchb.f., *D. satyriopsis*, *D. ukingensis* Schltr., *D. verdickii* De Wild., *D. welwitschii*, *D. zombica* N.E.Br., an unidentified *Disa* species and several samples that showed a highest percentage identity match with *D. robusta*, *D. satyriopsis* and *D. welwitschii.* The posterior probability for this clade is only 0.25, and combined with the posterior probabilities present on the within-species nodes, indicates a lot of uncertainty for this identification.

Overall, the most frequently encountered species were *Satyrium buchananii*, with 41 samples in eleven different collections, *Platycoryne crocea,* with 19 samples in three different collections, and *Disa robusta*, with 4–16 samples in two to seven different collections. *Myala* or original chikanda seems to contain *D. robusta*, *D. welwitschii* and *S. buchananii*. *Mbwelenge* or fake chikanda seems to correspond only to *S. buchananii* and *mshilamshila* to one or several *Brachycorythis* species. *Kasebelele* and *kapapa* referred to *Habenaria* spp., *P. crocea*, *S. carsonii* and *S. kitimboense*. The mixed collections contained all of the above-mentioned species as well as an unidentified *Habenaria* species. An overview of the local chikanda classification types, their collection numbers, identifications and number of samples can be found in Table 1.

## 4. Discussion

### 4.1. Species Used for Chikanda

Using DNA barcoding as an identification tool for chikanda tubers sold on local Zambian markets has allowed us to determine for the first time which orchid species are sold on local markets. Previous studies identifying orchids used for chikanda relied on voucher collections made with collectors in the field and their morphological identification, which requires a qualified orchid taxonomist [6,11,13,16,17]. Additionally, relying on local harvesters for details on chikanda collection might not always lead to collection in the areas where actual intensive harvesting is taking place, since some harvesters do not like to divulge where the best places to harvest are, and in some initial harvesting areas, such as the Kitulo Plateau in Tanzania’s Southern Highlands, collection is now prohibited [32]. The current study identified 16 orchid species present on the markets, including at least three previously undocumented ones: *Brachycorythis* cf. *friesii*, *Platycoryne crocea* and an unidentified species in a genus, which appears to be closely related to *Habenaria*, but is not present in our reference database.

Orchids used for chikanda seem to be harvested from several provinces in Zambia, as well as at least two regions in Tanzania. Moreover, three international chikanda trade hubs in towns on or close to the border with the DRC (Chililabombwe and Kasumbalesa) and Angola (Mwinilunga) were identified, in addition to the already known trade hub Tunduma-Nakonde on the Tanzanian border [7,11]. However, unlike what has been reported in other studies, no harvest from Malawi was mentioned by the people interviewed in this study, which could mean that trade from this country is currently not taking place. Another explanation is that this information is lost en route and that only Tanzania, being geographically closer and thus more easily accessible for the Bemba people, is mentioned as a region of origin for chikanda traded in Zambia.

### 4.2. DNA Barcoding Performance

Since the term DNA barcoding was coined in 2003 [19], a plethora of studies applying DNA (meta)barcoding has been performed ranging from retrieving orchids from paleoenvironments [33], preserved in mammoth dung [34], to the identification of Iranian orchid tubers used for salep [4]. In this study, a combined use of the core plant markers *matK* and *rbcL* and the nuclear ribosomal ITS region was used to attempt the species-level identification of tubers traded on Zambian markets. Although genetic distance calculations showed limited interspecific distances between closely related species for all three barcoding markers, DNA barcoding allowed for species-level identification for several of the frequently sold chikanda species. The data shows that the core land-plant DNA barcoding markers *rbcL* and *matK* were not suitable because of the limited variability between species (*matK* and *rbcL*), amplification problems (*matK*), and/or a limited sequence reference database (*rbcL*). Similar performance has been reported in other barcoding studies and indicates that the use of these core land plant markers might not be suitable for the analysis of samples with degraded DNA and for the identification of closely related species [35,36,37]. nrITS was shown to be more suitable as a barcode marker to distinguish between different chikanda species, but is not discriminative enough to enable reliable species level identification in certain orchid clades, such as the clade with *Platycoryne crocea*, *P. buchananiana* and *Habenaria buchananii*; the clade with *H. schimperiana*, *H. kyimbilae* and *H. microsaccos* and the clade containing *Disa* sp. 1. Another drawback of nrITS is the presence of multiple ITS paralogs in the ribosomal genome. Usually, these copies would show high similarities due to concerted evolution [38,39], but this is not always the case [40,41,42,43]. In our case, potentially different nrITS ribotypes became fixed in different orchid populations, and having only one of them in our reference database could lead to the unresolved identifications observed. Our bPTP results show that even nrITS has too low a resolution to reliably identify species with high posterior probability support using this method. It does demonstrate, however, how valuable the use of tree-based methods can be, since it shows the relations between the sequences and can be used to determine if some of the unidentified samples are likely to belong to the same species or a different species within the same genus. Even if no species-level identification can be made for these samples, it is possible to use the clustering to determine the diversity of species used. Although several samples can only be reliably identified as *Habenaria* sp., we find that they are likely to belong to at least three different species (*Habenaria* aff. *helicoplectrum*, *Habenaria* cf. sp. DO122 in the clade with *H. schimperiana*, *H. kyimbilae* and *H. microsaccos* and lastly the *Platycoryne* sp./*Habenaria* sp., which group together with *H. buchananii*, *P. buchananiana* and *P. crocea*. Expansion of the reference database, by including at least one individual per species, and preferably multiple individuals per species from different populations and countries, could ultimately solve the remaining challenges, and this seems the way forward for the identification of the traded chikanda tubers, as well as other species unidentifiable by morphology. Similar studies using DNA barcoding for the identification of unknown samples show comparable results for the employed barcoding markers. In a study on the identification of orchids used for salep, nrITS showed a sequencing success more than three times higher than *matK* as well as a two-fold higher species-level identification success [4]. Moreover, the similarity-based approach seemed to outperform the tree-based identification method (ML) in this study as well with 57% and 39% species-level identifications, respectively. nrITS also shows the highest identification performance in studies on the identification of medicinal plants [44,45], which supports the idea that it is advisable to add a more discriminative marker to the two core land-plant barcodes in studies where it is needed to distinguish between closely-related species. Moreover, our results stress the need for a phylogenetically underpinned taxonomic framework, which is currently available for *Disa* [46] and *Satyrium* [47], but not yet for *Habenaria* and related genera. A remaining limitation is the limited DNA extraction and amplification result, which could potentially be improved by experimenting with different extraction methods and primer pairs that would allow the amplification of segments of the *matK* marker [48,49,50].

### 4.3. Local Versus Scientific Classification of Chikanda

The results of our identifications of chikanda orchids traded in Zambia show that the local classification systems for chikanda are not congruent with the botanical classification of orchid species. The orchids sold on the markets were grouped according to area of origin, tuber consistency preference, or shape of the tuber, but often the tubers offered for sale were mixtures. The different local types of chikanda sometimes show a variation in orchid species that are identified within these local groupings. When we look at the chikanda type known as *kapapa*, for example, SJK44 contains *Platycoryne crocea*, whereas SJK11, which is supposed to be a mix of *kasebelela* and *kapapa,* only seems to contain *Satyrium* species. In the case of other chikanda types, there seems to be more consistency: *myala* or real chikanda referred to *Disa robusta*, *D. welwitschii* and *S. buchananii*, *mshilamshila* samples were identified as *Brachycorythis* species and *mbwelenge* or fake chikanda was made of *S. buchananii*. However, our previous study on the analysis of Tanzanian chikanda cakes showed that the cake made with fake chikanda tubers also contained *Disa miniata*, *Satyrium anomalum, S. comptum, S. elongatum, S. riparium, S. shirense* and *S. volkensii*, indicating that there might be some differences between fake chikanda samples as well [18]. It is well known in the literature that local species concepts are not necessarily congruent with scientific classifications and that species might be subject to over- or under-differentiation [51,52]. In this case, the grouping of the orchids according to the area of origin, shape or consistency preference or plainly under the general term chikanda is clearly a case of under-differentiation, as a much higher diversity was retrieved when employing DNA barcoding. In order to more reliably identify the orchid species used for a particular chikanda type, more samples per local classification need to be analyzed.

### 4.4. Orchid Availability and Conservation

In recent decades, chikanda has made a remarkable leap in popularity. The first record of chikanda use made by Audrey Richards [8] described the relish as a poor man’s food, eaten in times of famine. Recent studies, from 2002 onwards, show that chikanda has emerged as a Zambian snack which is popular throughout the country. Studies on chikanda report that, with this rise in popularity, the orchid harvest has escalated and is pressuring local Zambian orchid populations as well as those in neighboring countries [7,9,11,53]. Many people involved in the chikanda trade indicated that chikanda plants were becoming scarce, and many were concerned about both the quality as well as the quantity of the orchids available. Our study also confirmed a significant international trade network for chikanda sources in several regions in Tanzania, as well as in the Democratic Republic of Congo and Angola. In our current study, we found at least 16 different orchid species sold as chikanda on the Zambian markets and an overview of previous studies contains 46 species reported to be used for chikanda (Table S7). This brings us from the use of an initial two orchid species reported for chikanda [12] to a total of 49 species in eight different genera. The increased harvesting pressure, in combination with the indiscriminate harvesting and use of many more species than earlier assumed, poses a threat to nearly half of the terrestrial orchids occurring in these regions. Despite the establishment of Kitulo National Park in Tanzania, with orchid conservation as a prime concern, it seems that harvesting continues even there, since *iringe* tubers found in this study come specifically from this region [6,11]. Currently, there are only seven *Disa* species from Zambia and surrounding countries registered on the global IUCN Red List and no species from other genera used for chikanda [54]. Most of the orchid species used for chikanda seem to have a widespread distribution, but local populations as well as endemic species could be at risk of overharvesting, and we urge the addition of the most frequently traded chikanda species, such as *Disa robusta* and *Satyrium buchananii*, to the IUCN Red List. Although no reduction in commerce is evident, the people involved in the chikanda trade seem genuinely concerned about the welfare of local orchids and to be interested in exploring other options. Since the chikanda harvesters especially seem to be in a vulnerable position, where they have to rely on surrounding natural resources to secure their livelihoods [15], it is essential that, when trying to protect orchids used for chikanda, the situation of the people dependent on the trade be taken into account as well. Currently, the development of sustainable cultivation of chikanda orchids is being attempted in collaboration with the Cape Institute of Micropropagation (Barrydale, South Africa), and possible alternative sources of income for the people involved in chikanda trade, such as honey production, are being explored [55]. Alternatively, since the purpose of the chikanda orchids is mainly to bind and create an elastic structure for the cake, it might be possible to encourage the use of an alternative source of starch to replace the tubers.

## 5. Conclusions

DNA barcoding using the nuclear ribosomal ITS marker proved to be useful in identifying terrestrial orchid species traded as chikanda on local Zambian markets and outperformed identification using the core land-plant barcoding markers *matK* and *rbcL*. Sixteen orchid species, including three previously undocumented ones, were identified from marketed chikanda tubers, bringing the total number of orchid species used for chikanda to at least 49. The species most frequently found on the markets were *Disa robusta*, *Satyrium buchananii* and *Platycoryne crocea*. However, the results are only as good at the reference material, and an expanded reference database in combination with an underpinned phylogenetic framework for *Habenaria* and related genera would likely improve the reliability of the identifications. Tubers are harvested from various regions in Zambia and Tanzania, and additional international chikanda trade hubs have been identified on the border with the Democratic Republic of Congo and Angola. People involved in the chikanda trade indicate that both orchid quality and quantity are decreasing and are willing to consider alternatives to the chikanda trade to secure their income.

## Figures and Tables

**Table 1:** Overview of the different local chikanda classification types, their collections and the identified scientific species.

Vernacular Name	Collections	Reported Origin	Barcoding IDs	# Samples
Fungulwe	SJK16	unknown	*Disa robusta*	1
Iringe	SJK17	Tanzania	*Satyrium buchananii*	1
			*Satyrium carsonii*	1
John White	SJK39	Mporokoso, Zambia	*Satyrium buchananii*	1
Kabula seke	SJK46	Serenje, Zambia	*Habenaria* sp. (Clade *H*. *schimperiana*, *H*. *kyimbilae*, *H.* *microsaccos*	1
			*Platycoryne crocea*	7
Kapapa	SJK44	Mporokoso, Zambia	*Platycoryne crocea*	6
Kasebelela, John White and Myala	SJK41	Chinsali and Mporokoso, Zambia and Tanzania	*Habenaria* cf sp. DO122 (Clade *H*. *schimperiana*, *H*. *kyimbilae*, *H.* *microsaccos*	4
			*Platycoryne crocea*	4
			*Platycoryne* sp.*/Habenaria* sp.	2
Kasebulela and Kapapa	SJK11	Luwingu, Zambia	*Satyrium kitimboense*	6
			*Satyrium carsonii*	5
Mbwelenge	SJK5	Luwingu, Zambia	*Satyrium buchananii*	11
			*Satyrium* sp.	1
	SJK32	Serenje, Zambia	*Satyrium buchananii*	6
Mshilamshila	SJK7	Luwingu, Zambia	*Brachycorythis sp.*	1
	SJK12	Kawamba, Zambia	*Brachycorythis sp.*	1
			*Brachycorythis* cf. *friesii*	1
Myala	SJK4	Mwinilunga, Zambia;	*Disa robusta*	4
			*Disa welwitschii*	1
			*Satyrium buchananii*	4
	SJK18	Sumbawanga, Tanzania	*Disa robusta*	4
			*Satyrium buchananii*	1
Myala	SJK37	Kawambwa, Zambia	*Satyrium buchananii*	1
Myala and nampanda	SJK21	Luapula, Zambia	*Disa welwitschii*	2
			*Satyrium buchananii*	1
Ntonkonshi	SJK25	Democratic Republic of Congo	*Disa robusta*	1
Sumbawanga	SJK20	Sumbawanga, Tanzania	*Disa satyriopsis*	1
Mixed	SJK31	Serenje, Zambia	*Disa caffra*	1
			*Disa robusta*	1
			*Habenaria* cf sp. DO122 (Clade *H*. *schimperiana*, *H*. *kyimbilae*, *H.* *microsaccos*	1
			*Satyrium buchananii*	6
Unknown-mixed	SJK19	Luwingu, Zambia	*Satyrium carsonii*	1
			*Habenaria* aff. *helicoplectrum* (BB3151)	1
Unknown	SJK8	Mwinilunga, Zambia	*Disa miniata*	1
			*Disa robusta*	2
			*Disa welwitschii*	1
			*Satyrium buchananii*	2
	SJK9	Luwingu, Zambia	*Satyrium carsonii*	1
	SJK13	Kawamba, Zambia	*Disa celata*	1
			*Disa welwitschii*	1
			*Satyrium buchananii*	3

## Supplementary Materials

The following supplementary figures and tables are available online at http://www.mdpi.com/2073-4425/9/12/595/s1, Figure S1. bPTP analysis of all chikanda *matK* query and reference samples; query samples identifiers are the collection number and the BLAST identification; reference samples from GenBank have their 2-letter, 6-numeral accession numbers as identifier; other reference samples have their collection number and identification based on morphology as identifier. Figure S2. bPTP analysis of all chikanda *rbcL* query and reference samples; query samples identifiers are the collection number and the BLAST identification; reference samples from GenBank have their 2-letter, 6-numeral accession numbers as identifier; other reference samples have their collection number and identification based on morphology as identifier. Figure S3. bPTP analysis of all chikanda nrITS query and reference samples; query samples identifiers are the collection number and the BLAST identification; reference samples from GenBank have their 2-letter, 6-numeral accession numbers as identifier; other reference samples have their collection number and identification based on morphology as identifier. Table S1. Brahms RDE file of novel voucher specimens of orchid taxa samples in this study; for a thorough explanation of the column headers we refer to the online support material of the BRAHMS project [56]. Table S2. Genbank accession numbers of the chikanda tuber sequences. Table S3. Hit table with the first 5 BLAST top hits per sample for nrITS. Table S4. Hit table with the first 5 BLAST top hits per sample for *matK.* Table S5. Hit table with the first 5 BLAST top hits per sample for *rbcL.* Table S6. Successfully sequenced samples with their vernacular name, reported origin, identification based on sequence-similarity for nrITS and *matK*, identification based on the tree-based bPTP analysis and the consensus ID. Table S7. Orchid species used for chikanda according to literature.
