# Supplementary figures and images for: Trade in Zambian Edible Orchids—DNA Barcoding Reveals the Use of Unexpected Orchid Taxa for Chikanda
Source: Genes (Basel). 2018 Nov 30;9(12):595. doi: 10.3390/genes9120595 (PMC6315803; doi:10.3390/genes9120595)

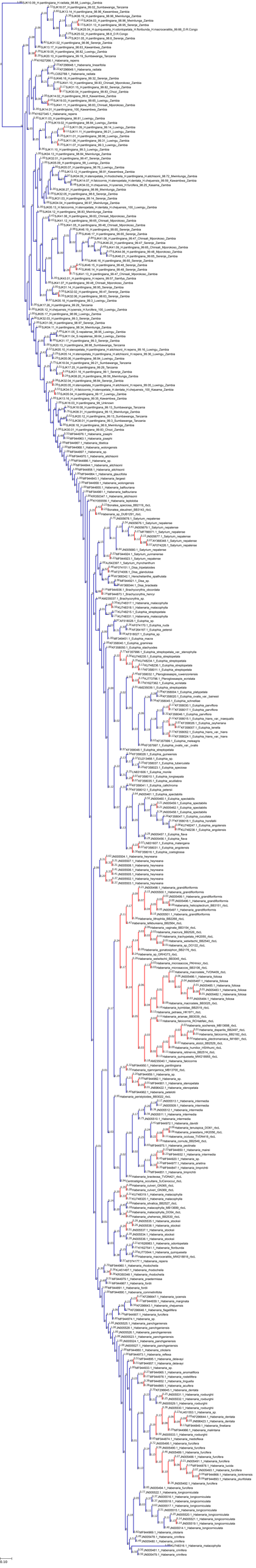

Supplement: Supplementary file 1 [file genes-09-00595-s001.zip › TiZeo_Supplementary_Files/Figure_S2_bPTP_rbcL.pdf]
