# Supplementary material for: Trade in Zambian Edible Orchids—DNA Barcoding Reveals the Use of Unexpected Orchid Taxa for Chikanda
Source: Genes (Basel). 2018 Nov 30;9(12):595. doi: 10.3390/genes9120595 (PMC6315803; doi:10.3390/genes9120595)

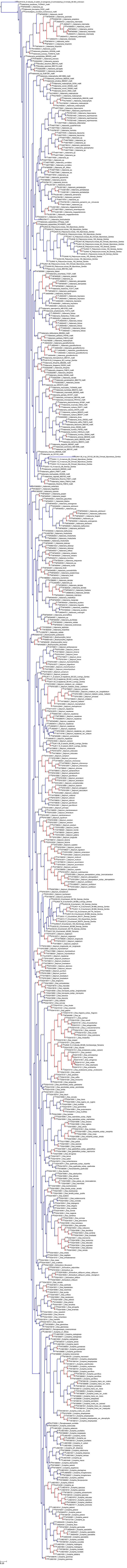

Supplement: Supplementary file 1 [file Figure_S1_bPTP_matK.pdf › TiZeo_Supplementary_Files/Figure_S1_bPTP_matK.pdf]
